# Unveiling the impact of ERAP1 and ERAP2 on migration, angiogenesis and ER stress response

**DOI:** 10.3389/fcell.2025.1564649

**Published:** 2025-03-28

**Authors:** Irma Saulle, Alessandra Velia Vitalyos, Daniel D’Agate, Mario Clerici, Mara Biasin

**Affiliations:** ^1^ Università degli Studi di Milano, Dipartimento di Scienze Biomediche e Cliniche, Milano, Italy; ^2^ Università degli Studi di Milano, Dipartimento di Fisiopatologia Medico-Chirurgica e dei Trapianti, Milano, Italy; ^3^ IRCCS, Fondazione Don Carlo Gnocchi, Milano, Italy

**Keywords:** ERAPs, cell biology, cell migration, ER stress, autopaghy

## Abstract

Recent studies have investigated the key roles exerted by ERAP1 and ERAP2 in maintaining cellular homeostasis, emphasizing their functions beyond traditional antigen processing and presentation. In particular, genetic variants of these IFNγ-inducible aminopeptidases significantly impact critical cellular pathways, including migration, angiogenesis, and autophagy, which are essential in immune responses and disease processes. ERAP1’s influence on endothelial cell migration and VEGF-driven angiogenesis, along with ERAP2’s role in managing stress-induced autophagy via the UPR, highlights their importance in cellular adaptation to stress and disease outcomes, including autoimmune diseases, cancer progression, and infections. By presenting recent insights into ERAP1 and ERAP2 functions, this review underscores their potential as therapeutic targets in immune regulation and cellular stress-response pathways.

## 1 Introduction

ERAP1 (Endoplasmic Reticulum Aminopeptidase) 1 and ERAP2 (together referred to as ERAPs) are two IFN (interferon)γ-inducible enzymes that belong to the oxytocinase subfamily of metallo-1 (M1) Zn^2+^ metallopeptidases, which are ubiquitously expressed in human tissues (Weimershaus et al., 2013). *ERAP* gene sequences are similar exhibiting 49% match and numerous gene polymorphisms (Stratikos and Stern, 2013). The tertiary crystal structure of ERAP1 consists of four different domains, which may exist in two diverse conformations: one open conformation that binds peptide, and one closed conformation containing a catalytic pocket able to initiate substrate hydrolysis (Kochan et al., 2011). ERAP1 can cleave nearly all peptide bonds, except those involving prolines, displaying variable efficiency contingent upon the N-terminal side chain of the substrate. It favors nonpolar residues while showing reduced efficacy with polar and charged ones. Additionally, it has a propensity to cleave peptides that are 9 to 16 amino acids, usually resulting in shorter fragments of about 8 to 9 amino acids (Chang et al., 2005). The overall structure and domain arrangement of ERAP2 are very similar to those of ERAP1, though specific structural features may contribute to its different substrate selectivity. Indeed, ERAP2 has an internal cavity with a distinct shape, characterized by a higher prevalence of hydrophobic residues with a notable preference for arginine. Furthermore, ERAP2 cleaves best 9-mers and shorter peptides most effectively, becoming progressively less efficient with longer ones (Mpakali et al., 2015).

ERAP1 and ERAP2 are primarily localized within the endoplasmic reticulum (ER) and play pivotal roles in the trimming of antigenic peptide precursors to the optimal length for binding to major histocompatibility complex (MHC) class I molecules (Kloetzel and Ossendorp, 2004; Mattorre et al., 2022). The MHC I/antigen complexes can then migrate to the cellular membrane to ensure optimal presentation to antigen-specific CD8^+^ T cells, thereby promoting defense against tumors, viruses, and other intracellular antigens. In this context, ERAP1 and ERAP2 can act as homodimers and/or can physically interact with each other to assemble into heterodimers (Chen et al., 2016; Li et al., 2019; Tedeschi et al., 2020). This interaction enables the integration of their enzymatic properties into a single complex, thereby boosting peptide-trimming efficiency and facilitating the presentation of a wider variety of antigens, ultimately enhancing the immunogenicity of the antigen repertoire.

Unexpectedly, recent findings indicate that: 1) following inflammatory stimuli, ERAP molecules may be secreted either as soluble proteins or as cargo inside extracellular vesicles (EVs) (Goto et al., 2014; Saulle et al., 2019); 2) beyond antigen presentation, ERAP1 and ERAP2 catalyze numerous other “non-canonical” functions in the intracellular and extracellular milieu (Cui et al., 2003a; Cui et al., 2003b; Goto et al., 2014; 2015; Saulle et al., 2021a). Both the generation of the antigen repertoire and the unusual functions displayed by ERAPs are highly influenced by polymorphisms in their coding sequences, significantly altering their enzymatic activity and/or rate of expression (Stamogiannos et al., 2015; López de Castro, 2018).

This review provides an overview of functional investigations conducted on ERAP1 and ERAP2, highlighting their broader role beyond antigenic peptide processing, with a focus on the direct and indirect effects exerted by these two aminopeptidases on different cellular pathways potentially affecting cellular homeostasis. In particular, we will review the findings associating ERAP activity and ERAP genetic variants with cellular migration, angiogenesis, autophagy, and the unfolded protein response (UPR).

### 1.1 *ERAP1 and ERAP2* gene polymorphisms and expression levels

*ERAP* genes are located on chromosome 5q15 in opposite orientations. The *ERAP1* gene spans 47,379 base pairs and includes 20 exons, while the *ERAP2* gene covers 41,438 base pairs and contains 19 exons (Cifaldi et al., 2012). Both genes are polymorphic; in particular, the *ERAP1* sequence exhibits over 40,000 single nucleotide polymorphisms (SNPs) in both the intron and exon regions. E. Reeves *et al.* showed the presence of 13 haplotypes (Hap) classified into three categories: efficient, hypoactive, or hyperactive, based on the extent of change in their capacity to produce specific antigenic epitopes (Reeves et al., 2013; Ombrello et al., 2015). Polymorphic amino acids are frequently located near the catalytic site (residues 346, 349), in the peptide binding site (residues 725 and 730), in interdomain regions, or in other positions that can affect the conformational rearrangements associated with the acquisition of the enzymatic activity (residues 528 and 575) (Reeves et al., 2013; López de Castro, 2018). The *ERAP1* polymorphism that has garnered the most attention is rs30187, which encodes the amino acid switch K528R in the human population. This polymorphism significantly affects enzyme activity by altering the kinetics of the conformational transition between the enzyme’s active and inactive states (Evnouchidou et al., 2011). Another SNP associated with this one, *rs27044*, which encodes the Q730E amino acid substitution, has been shown to correlate with changes in peptide length preferences and trimming specificity (Reeves et al., 2013). This is likely related to the involvement of this residue in substrate binding (Reeves et al., 2013). Additional *ERAP1* polymorphisms impact the expression level, rather than its structural configuration, potentially affecting disease onset or susceptibility. However, the robust linkage disequilibrium (LD) among various polymorphisms likely results in limited variability among individuals (López de Castro, 2018).

In contrast to *ERAP1*, non-synonymous alterations affecting the amino acid sequence of ERAP2 appear to be rarer; indeed, only 11,097 SNPs have been documented, so far (Zhang et al., 2020). In most populations, there is a balanced frequency of a specific polymorphism known as the *rs2549782* SNP (Andrés et al., 2010; Biasin et al., 2013). This SNP, encoding the K392N amino acid substitution, modulates the enzymatic activity and substrate specificity of the aminopeptidase (Evnouchidou et al., 2012). *In vitro* studies have demonstrated that the 392N variant exhibits much greater efficacy than its 392K counterpart in trimming hydrophobic N-terminal residues (Yao et al., 2019). This disparity arises from alterations in both the catalytic and binding sites of the N-terminal domain. Consequently, this SNP can introduce variability in antigen-processing activity among individuals carrying either variant of the enzyme (Yao et al., 2019). The *rs2549782* is in LD with an additional 11 SNPs, allowing for the identification of two Haps (Haplotypes) (HapA, HapB), which have been maintained at intermediate frequencies in almost all human populations (Evnouchidou et al., 2012). Of note, one of these SNPs, *rs2248374* (A/G), in HapB (G allele) leads to the transcription of a spliced form with an elongated exon 10 and two in-frame TAG stop codons, which is degraded by the nonsense-mediated decay (NMD) process (Andrés et al., 2010). As a consequence, individuals who are HapB homozygous—approximately 25%—do not synthesize the protein, while those who are HapA (A allele) homozygous present roughly 50% more enzyme compared to heterozygous HapA/B individuals. The equal occurrence of both haplotypes suggests that despite undergoing NMD, transcripts originating from HapB may still offer fitness advantages, particularly in certain contexts. Accordingly, recent analyses revealed that cells derived from subjects carrying HapB may transcribe two previously unknown short isoforms (ISO3, ISO4), which may result in protection from viral infections (Ye et al., 2018; Saulle et al., 2020a).

In a recent study, a polymorphism situated in the *ERAP2* promoter region*,* *rs7586269*, was linked with reverse changes in the expression of *ERAP1* and *ERAP2*. Specifically, reduced expression of *ERAP2* coincided with heightened expression of *ERAP1,* suggesting a concerted regulation of the expression of both genes by this SNP. However, unlike *rs2248374*, the minor allele frequency of *rs7586269* is relatively low in most populations (Paladini et al., 2018).

Alterations in *ERAP* gene expression levels may also be associated with the onset/progression of several diseases as well. In this frame, in 2013, Wang and colleagues reported for the first time that ERAP expression is at least partially dependent on p53 (Wang et al., 2013). By combining chromatin immunoprecipitation sequencing with gene expression analysis, the researchers demonstrated that p53 enhances ERAP1 expression by binding to its specific response element. Indeed, when p53 is silenced, ERAP1 protein levels are reduced, leading to a consequent downregulation of MHC I expression in the isogenic human colon carcinoma cell line HCT116. Likewise, in H1N1-infected A549 pulmonary epithelial cells, the authors demonstrated that p53 activation by H1N1, leads to ERAP1 upregulation and, in turn, to an increase in MHC I expression (Wang et al., 2013). Moreover, Xiaolei Zhou demonstrated that the pharmacological induction of p53 prompts the overexpression of both aminopeptidases and enhances the presentation of heterologous OVA peptide-SIINFEKL on the cell surface of A375-OVA cells. Induction of these genes was p53-dependent, as it was completely abolished in p53-null A375p53KO (Zhou et al., 2021). Overall, emerging research indicates that beyond apoptosis, cell cycle progression, senescence, and metabolism regulation (Vousden and Prives, 2009), p53 participates in a wider array of cellular functions, including those involving immune response mechanisms (Taura et al., 2008; Sanz et al., 2019).

*ERAP’s* polymorphisms/expression levels have been the subject of intense investigation due to their potential implications in various diseases and immune-related processes, as discussed in the following paragraph. Briefly, *ERAP1* variants have been extensively studied in the context of autoimmune diseases, such as ankylosing spondylitis (AS) and psoriasis, as well as in cancer susceptibility. Similarly, polymorphisms in *ERAP2* have been associated with diverse conditions, including inflammatory bowel disease and viral infections (Reeves et al., 2013; Stamogiannos et al., 2015). However, while qualitative and/or quantitative alterations in ERAPs have been extensively reported to affect the antigen processing and presentation pathway, thus shaping immune responses, only a few studies have correlated *ERAPs’* polymorphisms/expression levels to the different modulation of specific cellular processes such as migration, metastasis formation or autophagy, so far. Addressing this issue would provide additional insights into the cellular mechanisms through which ERAPs may orchestrate the onset/progression of different diseases.

### 1.2 The role of *ERAP* variants in human pathology

**FIGURE 1:**
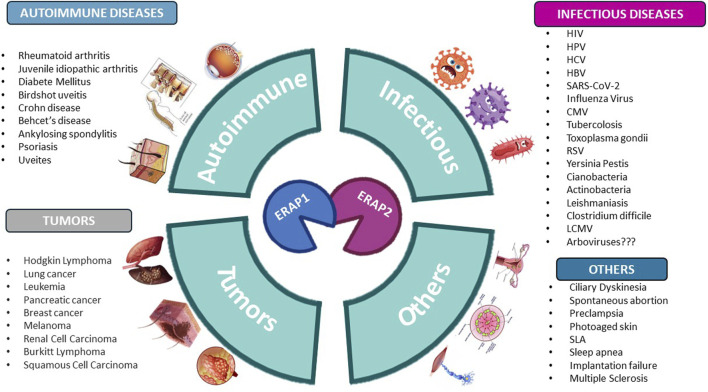
Human diseases correlated to variants and dysregulation of ERAP1 and ERAP2. Figure created with BioRender.com.

Since polymorphisms modify ERAP substrate specificity (Evnouchidou et al., 2012) and peptide-trimming kinetics (Evnouchidou et al., 2011), it is highly conceivable that the altered antigen processing by these enzymes is involved in disease pathophysiology. Indeed, variants and dysregulation of *ERAP1* and *ERAP2* have been strongly implicated in several pathological settings such as autoimmune diseases, cancer, infections, and inflammatory conditions, as summarized in Figure 1.

For instance, in autoimmune diseases, *ERAP1* variants *rs30187 (K528R)*, *rs27044 (Q730E)*, and *rs17482078 (R725Q)* have been associated with AS and Behçet’s disease (Reeves and James, 2018; Vancraenenbroeck et al., 2019). These variants affect the enzyme’s efficiency in trimming antigenic peptides to the optimal length required for MHC I binding, particularly in individuals with the HLA-B27 allele (Reeves and James, 2018). By modifying the peptide pool available for immune surveillance, these variants may lead to the presentation of self-peptides that trigger an autoimmune response. Likewise, in psoriasis, *ERAP1* variants *rs30187 (K528R)* and *rs2287987 (M349V)* contribute to inappropriate inflammatory responses through altered interactions with the HLA-C*06:02 molecule, a known psoriasis risk allele (Ryu et al., 2023). As for *ERAP2*, the *rs2248374* variant has been linked to both AS and Crohn’s disease (Paladini et al., 2018; Hamilton et al., 2023). Presumably, reduced ERAP2 activity can lead to incomplete peptide trimming, potentially affecting immune tolerance and promoting chronic inflammation.

Polymorphisms in the *ERAP1* and *ERAP2* genes have been associated with a range of cancers, as well, suggesting their involvement in tumor development and progression. The impacts of ERAPs in cancer pathophysiology appear to be complex, as these, can exert both positive and negative effects (York et al., 2006; 2006), depending on the specific cancer type. In some cases, ERAPs’ activity has been shown to enhance the immune system’s ability to recognize and eliminate cancer cells, as seen in thyroid (Compagnone et al., 2019) and colon cancers (Fruci et al., 2008); conversely, in other disease contexts, such as for melanoma and renal cell carcinoma, the *rs30187* and *rs27044 ERAP1* variants seem to be responsible for immune evasion, altering peptide trimming in ways that may reduce tumor cell visibility to CD8^+^ T cells, thus promoting progression and metastasis formation (Cifaldi et al., 2012; Stratikos, 2014).

As recently reviewed in Saulle et al., 2020b, key associations have also been found in infectious diseases like HIV, Hepatitis B, and Tuberculosis, where specific *ERAP1* and *ERAP2* polymorphisms appear to influence disease susceptibility and outcomes. ERAP2 has also been implicated in historical survival outcomes from the Black Death (bubonic plague) in Europe, revealing insights into how ancient selective pressures shaped modern immune responses (Klunk et al., 2022). The Black Death, caused by *Yersinia pestis*, was one of the deadliest pandemics, and evidence suggests that the *ERAP2* *rs2248374* variant provided a survival advantage against this pathogen (Klunk et al., 2022).

The extensive involvement of *ERAP1* and *ERAP2* genetic variants across a wide range of seemingly unrelated pathological conditions can be readily understood when considering their fundamental role in immune system modulation and antigen processing (de Castro and Stratikos, 2019; Babaie et al., 2020; Martín-Esteban et al., 2022). By fine-tuning the peptides available for presentation on MHC I molecules, *ERAP* genetic variations modulate immune responses, tolerance, and activation, which in turn affect susceptibility and progression of diverse immune-related and inflammatory disorders (Hanson et al., 2018; Saulle et al., 2020b; Saulle et al., 2021b). However, as reviewed in the following paragraphs, beyond their role in antigen processing, ERAP1 and ERAP2 partake in many different aspects of cellular homeostasis, including processes like the ER stress response, UPR, cellular migration, and angiogenesis (Mattorre et al., 2022; Evnouchidou et al., 2023). These unconventional mechanisms may, at least partially, rely on the release of soluble ERAPs into the extracellular milieu following inflammatory stimuli. Indeed, plasma levels of ERAP1 and ERAP2 have been recently reported as markers of disease severity in inflammatory bowel diseases, preeclampsia, ankylosing spondylitis, and hepatitis B infection (Ferreira et al., 2021; Liu et al., 2022; Bai et al., 2024; You et al., 2024). This awareness suggests that their contribution to disease susceptibility/progression may extend far beyond immune modulation alone. Notably, these additional roles may underpin their significant contributions to disease onset and severity, highlighting their potential as therapeutic targets across diverse pathological conditions.

## 2 ERAPs role in cellular homeostasis

As anticipated, recent research highlights that ERAP1 and ERAP2 in modulating cellular homeostasis are as important as their contributions to the antigen presentation pathway (López de Castro, 2018; Woon and Purcell, 2018). These enzymes manage protein quality control in the ER, helping to prevent peptide accumulation that can lead to ER stress and trigger the UPR (Senft and Ronai, 2015). Additionally, ERAP1 and ERAP2 influence cellular functions like migration and angiogenesis (Babaie et al., 2020). By modulating chemotactic signals and bioactive peptides, such as angiotensin II, they impact immune cell movement, tumor metastasis, and blood vessel formation (Mattorre et al., 2022). This broader functionality underscores the significance of ERAP1 and ERAP2 in key processes affecting not only immune responses but also cellular stability and disease progression—concepts that will be explored further in the following paragraphs.

### 2.1 Highlights on cell migration and angiogenesis

Cell migration is a fundamental biological process essential for multicellular organization, from embryonic development to adulthood (Trepat et al., 2012). It includes various steps, from large-scale epithelial migration during gastrulation to precise single-cell movements in tissue development, such as in the nervous system (Cooper, 2013; Jalise et al., 2024). In adults, it plays crucial roles in immune responses, wound healing, and tissue homeostasis (Guak and Krawczyk, 2020; Laaker et al., 2023). Cell migration is closely linked to angiogenesis—the formation of new blood vessels from pre-existing ones (Nelson, 2003). This process is regulated by chemotactic, apoptotic, and mechanotactic stimuli and involves extracellular matrix degradation and cytoskeletal remodeling (Carmeliet and Tessier-Lavigne, 2005; Lamichhane and Samir, 2023). Central to this process is vascular endothelial growth factor (VEGF), a key signalling molecule that stimulates endothelial cell migration and proliferation. VEGF is produced by various cells, including tumoral cells, and acts as a potent chemoattractant for endothelial cells, guiding their migration toward areas of higher VEGF concentration. Dysregulated cell migration lies at the crux of various diseases, from cancer metastasis to neurological disorders. While normal cells follow standard migration mechanisms, cancer cells exhibit a promigratory dominance without counteracting signals, enabling invasive phenotypes. Identifying highly expressed motility genes could serve as biomarkers for tumor progression and metastasis (Yamaguchi et al., 2005).

Living cells detect and respond to environmental cues, such as chemoattractant gradients and electric fields, guiding migration. In complex gradients, cells navigate via a stepwise approach for sustained directional movement. From a mechanistic standpoint, cells utilize diverse tactics that are contingent upon the mode of migration and cell type involved. For instance, bacterial cells utilize rotating helical filaments to propel themselves through fluids, while leukocytes employ a five specific stage process to navigate through tissues (Merino-Casallo et al., 2022).

1) Cellular polarization driven by external signals. Essential for directional migration, cell polarity requires asymmetry between the front and back. Key signaling molecules, including PI3Ks, PTEN, and phosphoinositides, contribute to this process (Haga and Ridley, 2016).
2) Extension of protrusions at the cell’s leading edge. Migration begins with lamellipodia or filopodia formation, driven by actin polymerization via the Arp2/3 complex. Rac and Cdc42 regulate this process, facilitating ECM adhesion (Warner et al., 2019).
3) Adhesion to the ECM. Migration relies on adhesion complexes that stabilize protrusions, provide traction, and transmit forces. Integrins play a key role in linking the ECM to the actin cytoskeleton (Karamanos et al., 2021).
4) Contraction of the cytoplasm to move the entire cell forward. The cytoskeleton, composed of actin filaments and microtubules, governs protrusion, adhesion, and retraction. Microtubules promote polarity, front protrusion, and rear retraction, facilitating migration (Murrell et al., 2015; Conboy et al., 2024).
5) Adhesion disassembling at the cell’s rear, enables the completion of the migratory cycle (Haga and Ridley, 2016; Kadzik et al., 2020).

Understanding migration mechanisms and unknown determinants can illuminate cellular behavior and offer therapeutic potential for diseases linked to abnormal cell movement, such as tumors. Among these factors, ERAPs have been repeatedly reported to directly and indirectly partaking in the regulation of cellular migration in different pathological settings, although results have not always been univocal.

### 2.2 ERAPs’ role in cell migration, angiogenesis and metastasis formation

The first report linking ERAP1 and ERAP2 expression to cell migration dates back to 2002. Miyashita and colleagues found that ERAP1 expression occurs in endothelial cells both during *in vitro* differentiation and *in vivo*, mainly in the angiogenesis region induced by VEGF (Miyashita et al., 2002). Accordingly, blocking ERAP1 expression by antisense oligodeoxynucleotide in endothelial cells led to the suppression of VEGF-induced migration, proliferation, and neo-vessel formation *in vitro*, along with attenuated angiogenesis *in vivo*. All of these functions, indeed, seem to hinge on ERAP1’s catalytic activity (Miyashita et al., 2002). Yamazaki *et al.*, for example, unveiled that ERAP1 can bind to phosphatidylinositol-dependent kinase 1 (PDK1), cleaving nine amino acids from its N-terminus. This cleavage event enables the association of S6 kinase (S6K) with 3-Phosphoinositide-dependent protein kinase 1 (PDK1) and ERAP1 upon VEGF stimulation, thus promoting the G1/S transition phase and boosting the cell cycle progression of endothelial cells (Yamazaki et al., 2004). An intriguing observation is that a mutant of the ERAP1 active site, devoid of enzymatic activity, exhibits a dominant-negative effect. These mutants form a complex with PDK1 but lack the ability to trim the requisite N-terminal amino acids, thereby preventing the association or activation of S6K. This dominant-negative impact results in reduced angiogenesis and has been shown to halt tumor growth, providing significant evidence for ERAP1’s role in cancer cell cycle regulation (Yamazaki et al., 2004).

In a similar study, it was found that ERAP1 modulates the spreading of endothelial cells by activating focal adhesion kinase (FAK) and endothelial integrins. Their activation enhances endothelial cell adhesion to the extracellular matrix through the enzymatic activation of Ras homolog gene family member A (RhoA), a Guanosine Triphosphatase (GTPase) protein responsible for actin cytoskeleton reorganization and cell body locomotion (Zebda et al., 2012). In this frame, Akada *et al.* also observed that ERAP1 suppression results in a significant inhibition of the murine endothelial cell line MSS31 spreading, which is secondary to a diminished activation of endothelial integrins, essential for cell adhesion (Akada et al., 2002). Conversely, in another study, Watanabe *et al.*, revealed that ERAP1 modulates VEGF-induced angiogenesis and endothelial cell migration in human endometrial carcinoma by controlling the renin-angiotensin system (RAS) through a dose-dependent cleavage of Angiotensin II (Ang II) (Watanabe et al., 2003). Further confirming this assumption, the migration of umbilical vascular endothelial cells (HUVECs) induced by Ang II treated-cell medium was abrogated with conditioned media from ERAP1-overexpressing cells (Watanabe et al., 2003). Moreover, in animal studies, ERAP1-overexpression by endometrial carcinoma cells leads to a reduction in both VEGF immunoreactivity and the number of blood vessels within tumors, implicating ERAP1 in the modulation of endothelial cell migration and VEGF-induced angiogenesis (Mehta et al., 2015). Beyond endothelial cells, ERAPs were recently demonstrated to trigger migration of immune cells, specifically neutrophils, as well (Saulle, et al., 2025).

An indirect correlation between ERAP1 and angiogenesis was established by network pharmacology, an approach for multi-target drug discovery based on bioinformatics and system biology (Akada et al., 2002). Specifically, this strategy was employed to investigate the comprehensive mechanism of total glucosides of peony (TGP) for rheumatoid arthritis (RA) treatment, pointing to ERAP1 as one of the hub TGP’s angiogenesis regulators in this chronic autoimmune disease (Batool et al., 2022).

More recently, Bufalieri *et al.*, indicated ERAP1 as a previously unidentified regulator of the Hedgehog (Hh) cascade, which plays a critical role in cell migration, proliferation, and differentiation, particularly during embryonic development and tissue homeostasis (Bufalieri et al., 2019). In particular, ERAP1 was demonstrated to catalyze beta-transducin repeat containing E3 ubiquitin protein ligase (βTrCP)-degradation by directly interacting with the βTrCP-bound deubiquitylase enzyme USP47. This interaction shields Gli transcription factors from βTrCP-mediated degradation, consequently enhancing unchecked cell migration, proliferation, differentiation and tumorigenesis, primarily in medulloblastoma, an extremely aggressive pediatric cancer (Reig et al., 2014). Understanding the precise mechanisms by which ERAP1 influences these cellular behaviors within the Hh pathway holds potential for therapeutic interventions targeting Hh-driven tumors.

Still, within the field of oncology, ERAP1 downregulation was demonstrated to be an independent prognostic parameter for the presence of lymph-node metastases in cervicovaginal carcinoma (Mehta et al., 2008). Furthermore, a meticulous investigation revealed significantly elevated levels of ERAP2 in oral cavity squamous cell carcinoma (OSCC) tissues compared to adjacent non-cancerous epithelial tissues. Functional assays demonstrated that ERAP2-knockdown inhibited the migration and invasion abilities of OSCC cells, with reductions of approximately 83% and 48%, respectively. As patients with ERAP2 overexpression exhibited poorer overall survival rates, these findings collectively highlight the link between ERAP2 overexpression, cervical metastasis, and a bleak prognosis for OSCC. Additionally, they propose the targeting of ERAP2 as a potential therapeutic approach to impede the progression and metastasis of OSCC (Kuo et al., 2017).

Likewise, ERAP2-expression has been repeatedly associated with pancreatic cancer prognosis and diagnosis (Wu et al., 2020; Li et al., 2021; 2022; Liu et al., 2021; Guan et al., 2022; Yu et al., 2022; Zhou et al., 2022; Chen et al., 2023). From a functional viewpoint such consistent interdependence was partially associated with ERAP2-dependent UPR-mediated autophagy (Guan et al., 2022), as further discussed in a dedicated paragraph. However, ERAP2-induced cellular migration and metastasis formation seem to be equally relevant in the onset and progression of pancreatic cancer (Yu et al., 2022). Indeed, Yu and colleagues observed exceptionally higher ERAP2 levels in pancreatic tumors compared to normal tissues, which were associated with poor prognosis in pancreatic cancer patients. Notably, inhibiting ERAP2 through knockdown techniques not only suppressed tumor growth but also augmented the efficacy of gemcitabine, frontline chemotherapy for pancreatic cancer, inhibiting cancer cell proliferation, migration, and invasion. These findings shed light on the pivotal role of ERAP2 in promoting pancreatic cancer progression, metastasis, and resistance to gemcitabine treatment (Yu et al., 2022).

Since, as previously discussed, *ERAPs* are highly polymorphic in individuals, it is imperative to conduct additional studies to ascertain whether these variations impact cellular migration, angiogenesis, and the formation of metastases. Specifically, it will be extremely interesting to assess whether subjects who are natural knockouts for *ERAP2* expression, because of the *rs2248374* polymorphism, display a different susceptibility/progression to OSCC or pancreatic cancer.

**FIGURE 2:**
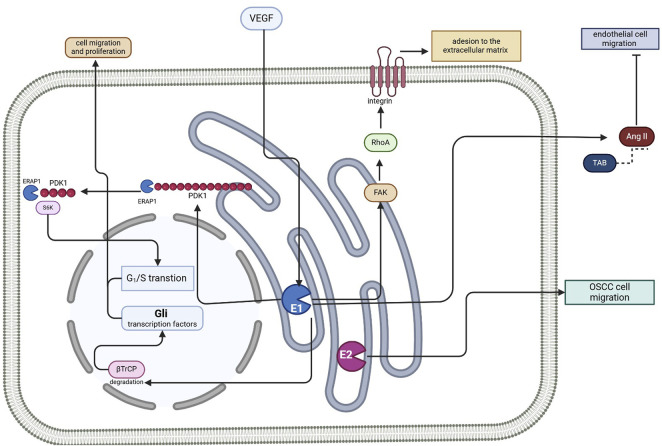
Schematic representation illustrating the multifaceted roles of ERAP1 and ERAP2 in cellular migration, angiogenesis, and metastasis formation. AngII: angiotensin II; βTrCP: F-box/WD repeat-containing protein 1A; E1: ERAP1; E2: ERAP2; FAK: focal adhesion kinase; OSCC: oral cavity squamous cell carcinoma; PDK1: pyruvate dehydrogenase kinase 1; RhoA: Ras homolog family member A; VEGF: vascular endothelial growth factor; S6K: S6 kinase; Figure created with BioRender.com.

Overall, these studies confirm ERAP participation in the migration process, enabling the movement of cells involved in both angiogenesis and metastasis formation (Figure 2). As all these characteristics are essential in maintaining the growth of solid tumors, it will be valuable to consider the targeting of ERAPs as a potential therapeutic approach to impede angiogenesis, tumor progression and metastasis formation, thereby facilitating personalized treatment approaches in their management.

### 2.3 Highlights on endoplasmic reticulum (ER) stress response and autophagy

Cellular stress, induced by external or internal cues, such as the accumulation of misfolded proteins, calcium imbalance, oxidative stress, and nutrient deprivation, triggers well-orchestrated processes to restore cellular homeostasis or commit to cell death (Senft and Ronai, 2015; Lamichhane and Samir, 2023). These pathways include the UPR, ER-associated degradation (ERAD), and autophagy, contributing to the ER stress response. UPR restores ER homeostasis by reducing protein synthesis, enhancing protein folding, and eliminating misfolded proteins. It involves three key ER transmembrane sensors: PERK, ATF6, and IRE1, whose activity is controlled by Binding Immunoglobulin Protein (BiP) (Read and Schröder, 2021; Lamichhane and Samir, 2023). To alleviate ER burden, BiP dissociates from these sensors, activating them. IRE1 oligomerizes, autophosphorylates, and splices XBP1 mRNA to produce XBP1s, which translocates to the nucleus and upregulates genes involved in ERAD, protein folding, and ER expansion. PERK dimerizes and phosphorylates eIF2α, attenuating global translation. Phosphorylated eIF2α increases ATF4 mRNA translation, inducing genes for antioxidant responses and apoptosis. ATF6 translocates to the Golgi for cleavage by Site-1 and -2 proteases (S1P, S2P), allowing nuclear entry to induce ER chaperones and ER stress response genes, including XBP1 (Read and Schröder, 2021; Lamichhane and Samir, 2023).

When the mechanisms to restore ER homeostasis fail, other stress-response pathways, such as autophagy (specifically macroautophagy), can be initiated (Rashid et al., 2015). This process targets dysfunctional proteins, damaged organelles, and intracellular pathogens. Macroautophagy relies on more than 40 autophagy-related (ATG) proteins (Rashid et al., 2015). It may be induced following Ca2+ release, secondary to ER stress, activating the ULK1 complex, which integrates nutrient starvation signals and promotes phagophore nucleation. After nucleation, two ubiquitin-like conjugation systems, ATG12-ATG5-ATG16L1 and LC3 lipidation, manage phagophore extension (Mizushima et al., 2003). ATG4 cleaves pro-LC3 to form LC3-I, which conjugates to phosphatidylethanolamine (PE) via ATG7, generating LC3-II. LC3-II is recruited to the autophagosome membrane, driving elongation (Tanida et al., 2004). Autophagy receptors like p62/SQSTM1 recognize ubiquitinated cargo and recruit it to the autophagosome via LC3-II (Kumar et al., 2022). Autophagosomes migrate along microtubules, fusing with late endosomes and/or lysosomes to form autolysosomes. Fusion is mediated by Rab GTPases and SNARE proteins (Zhao and Zhang, 2019). Termination involves ATG4-mediated LC3-II cleavage and lysosomal efflux pump export of degradation products for reuse, generating energy and new cellular components (Chen et al., 2019).

ER stress and autophagy are closely linked, interacting through various molecules. UPR signaling via PERK, IRE1α, and ATF6, along with CHOP, upregulates autophagy genes, including ATGs and LC3 (Verfaillie et al., 2010; Kwon et al., n. d.). PERK phosphorylates p62, while IRE1α and XBP1s upregulate Beclin-1 transcription, promoting autophagy (Song et al., 2018). ATF6 upregulates DAPK1, which phosphorylates Beclin-1, leading to the dissociation of the autophagy inhibitor Bcl-2 (Fujiwara et al., 2016).

However, depending on the magnitude and duration of ER stress and cell type, the UPR/autophagy pathway may also induce apoptosis (Song et al., 2018).

### 2.4 ERAPs’ role in ER stress response and autophagy

The correlation between ERAP1 and ERAP2 proteins with ER homeostasis and autophagy is strictly dependent on their cellular localization within the ER and their enzymatic activity. Indeed, polymorphisms in *ERAP* gene sequences can affect their enzymatic function and specificity, limiting the production of correct-sized peptides capable of binding to MHC class I molecules (Evans et al., 2011; Evnouchidou et al., 2011; Reeves et al., 2013; Mpakali et al., 2015; Stamogiannos et al., 2015). This, in turn, may lead to the accumulation of over-trimmed/under-trimmed peptides, depending on the specific polymorphism, as well as empty MHC-I heavy chains in the ER, causing ER stress and UPR activation (Colbert et al., 2009). This specific phenomenon has been extensively examined in an attempt to explain the strong genetic association between ERAPs, HLA-B27 and AS (Vitulano et al., 2017; Paladini et al., 2019). HLA-B27 is prone to slow folding and accumulates as a misfolded protein in the ER, thus increasing ERAD activity, UPR and autophagy, especially during inflammation, when HLA-B27 production is elevated (Navid et al., 2018; Busch et al., 2019). Moreover, misfolded HLA-B27 molecules display the unique characteristic of forming free heavy chain (FHC) dimers; (Mear et al., 1999); these dimers, on one hand, may be retained at the intracellular level, thus activating the UPR, ER stress, and the release of pro-inflammatory cytokines (IL-17, IL-23, IFNγ) (Pedersen and Maksymowych, 2019); on the other hand FHCs may be expressed on the cell surface, potentially being recognized by CD4^+^ T cells or NK, thereby triggering abnormal immune responses (Evans, 1999; Garcia-Montoya et al., 2018; Grandon et al., 2019). Accordingly, in bone marrow-derived macrophages (BMDMs) from a transgenic rat model overexpressing misfolded HLA-B27 proteins, mRNA expression of BiP, CHOP, and XBP1, as well as pro-inflammatory cytokines was significantly increased, suggesting UPR activation with possible implications for human diseases (DeLay et al., 2009). In this context, *ERAP1* AS-risk variants display higher enzymatic activity and/or expression, which lowers the surface expression of HLA-B27–peptide complexes by removing antigens that typically bind to HLA-B27 (Kenna et al., 2015b; Reeves and James, 2018). The same conclusions were drawn by Wang and colleagues while investigating the effects of ERAP1 variations on the ER stress-autophagy-inflammation axis. Indeed, they demonstrated that PBMCs isolated from Taiwanese AS patients carrying the *ERAP1*-001 haplotype, leading to ERAP1 overexpression, displayed higher production of FHCs and FHC dimers, UPR (BiP, CHOP and XBP1), autophagy (Beclin-1, LC3 I and LC3 II) and inflammatory (caspase 1 and IL-1β) markers, compared to *ERAP1*-002 homozygous donors, who produce lower ERAP1 quantities. Of note, analogous results were recapitulated in *ERAP1*-001-transfected U937 cells, suggesting that ERAP1-overexpression activates in a cascade: i) abnormal peptide processing; ii) misfolding of HLA-B27 complexes; iii) UPR; iv) and autophagy, which, by reducing ER stress, could provide cytoprotection (Wang et al., 2022). Further supporting this theory, in HLA-B27 transgenic rats, Tran *et al.* observed an increased folding of this MHC class I molecule following *ERAP1* knockdown, which in turn was associated with lower intracellular accumulation of FHC dimers as well as BiP, CHOP, and XBP1 expression suggestive of reduced UPR (Tran et al., 2023). However, this connection linking *ERAP1* silencing, UPR, and AS does not fully align with previous studies. For example, by studying the impact of natural *ERAP2* deficiency (rs2248374) on HLA-B27 expression in PBMCs from AS patients, it was demonstrated that this spontaneous knockdown causes an increased MHC class I FHC expression and the upregulation of the UPR pathway, assessed through CHOP and BiP quantification (Zhang et al., 2017). Likewise, Blake and colleagues demonstrated that BMDMs and CD4^+^ T cells isolated from *ERAP1* ^−/−^ mice display an exaggerated ER stress response, associated with an increased expression of ER stress-associated genes (BiP, XBP1s, IRE-1α, and p-IRE1α), due to lack of normal ERAP1 functions (Blake et al., 2022). Kenna and colleagues hold a further different viewpoint; indeed, they did not find associations among either AS-associated rs30187 *ERAP1* genotypes or rs2248374 *ERAP2* genotypes, HLA-B27 status, and ER stress (Kenna et al., 2015b; Robinson et al., 2015). Specifically, they reported that the expression levels of the ER stress markers GRP78 (8 kDa glucose-regulated protein) and CHOP in PBMCs and ileal biopsies from HLA-B27 AS patients were independent of *rs30187 ERAP1* variant or *rs2248374 ERAP2* variant. They, therefore, concluded that aberrant ERAP1 activity or ERAP2 absence does not affect ER-stress levels in AS and that ERAP1/ERAP2 and HLA-B27 influence disease susceptibility through other mechanisms (Kenna et al., 2015a; Robinson et al., 2015). The role of ERAPs in autophagy has been further substantiated in immune cells. Specifically, the administration of 300 ng of recombinant ERAP1 and/or ERAP2 proteins, mimicking stressor-induced secretion, boosts autophagy process in neutrophils. This was evidenced by increased LC3b levels and enhanced degradation of p62. These findings highlight the significance of ERAP activity in modulating ER stress and the autophagic response, particularly in innate immune cells (Saulle et al., 2025).

Notably, in all of these studies, ERAP-expression levels and/or alterations in their enzymatic activity seem to correlate with the maintenance of ER homeostasis. However, in a recent paper by Thomaidou and colleagues, this concept was somewhat overturned, suggesting that it is the increased cellular stress status, as measured by XBP1 splicing, that promotes the post-transcriptional upregulation of ERAP1 expression (Thomaidou et al., 2020). In particular, by investigating the role of ERAP1 in the aetiopathogenesis of type 1 diabetes in human β-cells, the authors demonstrated that IRE1α inhibition correlates with the upregulation of miR-17, which negatively affects ERAP1 expression via direct interaction with its 3′-UTR region, ultimately reducing pre-pro-insulin processing. (Thomaidou et al., 2020). These findings demonstrate a direct link between ER stress, ERAP1, and β-cell death, emphasizing the important role of ER, and possibly ERAP1, modulators in shaping antigenic insulin peptides for autoimmune diabetogenic CTLs.

More recently, Guan *et al.* demonstrated a strong correlation between pancreatic stellate cell (PSC) activation, autophagy and ERAP2 (Guan et al., 2022). In particular, the authors demonstrated that *ERAP2* silencing in PSC promotes a quiescent state and a drastic reduction of UPR-mediated autophagy as assessed through calnexin, IRE1α, PERK, the LC3II-lipidated form, and p62 degradation analyses. In addition, *ERAP2* knockdown decreased the PSC’s ability to promote migration and the invasion of pancreatic ductal adenocarcinoma (PDAC) by inhibiting ER-derived autophagy, and, *in vivo* xenografted tumor growth and fibrosis. Overall, these findings demonstrate that ER stress and the resulting UPR signaling pathway trigger ERAP2-dependent autophagy and PSC activation, uncovering the benefits of targeting ERAP2 as a potentially promising strategy for the treatment of pancreatic cancer (Guan et al., 2022).

Further reinforcing ERAP involvement in the loop of ER stress, in 2005 it was demonstrated that both peptidases localize to a large extent with calnexin (Saveanu et al., 2005), an ER chaperone involved in the quality control of protein folding (Schrag et al., 2001). Following ERAP2 knockdown calnexin expression was significantly suppressed (Guan et al., 2022). In addition, both ERAP1 and ERAP2 showed an enhanced localization in autophagic vesicles upon proteasome inhibition (Limanaqi et al., 2024). Last but not least, Palu *et al* identified *ERAP1/2* as orthologous to *the superdeath Drosophila melanogaster* gene (Palu et al., 2020). The loss of *superdeath* activity diminishes apoptosis and degeneration without interfering with the activation of ER stress sensors IRE1 and PERK. While these sensors can initiate apoptosis under prolonged stress, they are also crucial for cell survival and restoring cellular homeostasis (Sano and Reed, 2013). *Superdeath* loss maintains these survival functions while reducing Jun N-terminal kinases (JNK) signalling via CDK5 activation (Palu et al., 2020). Therefore, the authors suggest that targeting *superdeath* orthologs could be a promising therapeutic strategy to promote cell survival in degenerative diseases.

**FIGURE 3:**
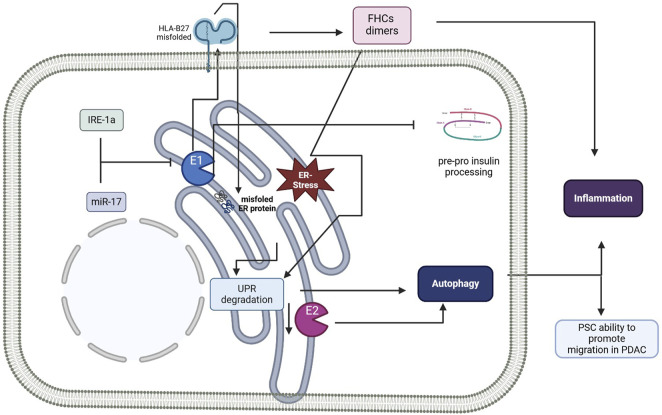
Schematic representation summarizing the role of ERAPs in the unfolded protein response (UPR) and autophagy. E1, ERAP1; E2, ERAP2; ER, endoplasmic reticulum; HLA-B27, human leucocyte antigen-B27; FHC, free heavy chain; IRE-1a, serine/threonine-protein kinase/endoribonuclease inositol-requiring enzyme 1 α; PSC, pancreatic stellate cell; PDAC, pancreatic ductal adenocarcinoma; UPR, unfolded protein response. Figure created with BioRender.com.

As a whole, the reported studies suggest controversial findings and nuanced crosstalk between ERAPs/ER stress/autophagy (Figure 3). In general, it seems that the role of ERAP is far more associated with the correct folding and assembly of MHC class I molecules than the generation of specific peptides as antigenic determinants. However, to consider the therapeutic possibility of targeting ERAPs to modulate ER stress in different pathological conditions, further analyses is mandatory for a deeper comprehension of the molecular mechanisms governing the ERAPs/ER stress axis.

## 3 ERAP1 and ERAP2 inhibitors: advances and therapeutic potential

ERAP1 and ERAP2 play crucial roles in the modulation of the immune response, making them attractive targets for therapeutic intervention, especially in autoimmune diseases. Researchers have made significant strides in developing inhibitors for these endoplasmic reticulum aminopeptidases, which are essential for antigen processing and presentation. Initial efforts yielded broad-spectrum inhibitors that affected both ERAP1 and ERAP2, presenting challenges due to potential unintended effects on immune modulation. As recently reviewed by Fougiaxis and colleagues, approximately 15 chemical series of ERAP inhibitors have been reported (Fougiaxis et al., 2024). Most of them (61%) were rationally designed, while others were discovered through high-throughput screening and *in silico* strategies (Guzanov, 2025). Notable chemical series include phosphinic peptidomimetic inhibitors and structure-based designed diaminobenzoic acids, which target the catalytic site. Additionally, allosteric inhibitors have been identified, revealing modulation pockets specific to ERAP1 (Fougiaxis et al., 2024). This perspective highlights the key inhibitors, focusing on potency and selectivity, discovery strategies, and binding modes. The first rationally designed ERAP inhibitors were produced by Zevroudi and colleagues, namely, DG002 and DG013 (Zervoudi et al., 2013). They demonstrated to reduce the expression of HLA-ABC and a consequent cytotoxic CD8 T response (Zervoudi et al., 2013), in addition, Chen and colleague, showed a drastic decrease HLA-B27 free heavy chain in HeLa-B27, the differentiation of Th17 cells, and the secretion of IL-17A from CD4^+^ T cells (Chen et al., 2016). One of non-rationally design inhibitors is Bestatin that is currently being evaluated in clinical trials in oncology (Vourloumis et al., 2022). Bestatin was later shown to be also a micromolar inhibitor of ERAP1 and a weak inhibitor of ERAP2 and Insulin Regulated Aminopeptidase (IRAP), another M1 zinc metalloproteases refining the peptidome in the endosomes (Salomon et al., 2018; Vourloumis et al., 2022). Thimerosal, identified through virtual screening, shows submicromolar inhibition only for ERAP1 but does not affect ERAP2 or IRAP. Additionally, thimerosal has a dose-dependent effect on antigen presentation in bone marrow-derived dendritic cells (BMDCs) that are treated with ovalbumin and exposed to OT-I CD8^+^ T cells. This effect was demonstrated to be specifically linked to ERAP1, as thimerosal does not produce any activity in BMDCs lacking ERAP1 (Stamogiannos et al., 2016).

Recent advancements have led to the design of highly selective inhibitors, like GRWD5769 for ERAP1, currently in clinical trials to enhance immune responses against tumors by promoting neoantigen presentation (Trial EMITT-1, 2025). Conversely, research on selective ERAP2 inhibitors remains limited, with some existing compounds showing insufficient potency and selectivity (Fougiaxis et al., 2024; Trial EMITT-1, 2025). Although many of the phosphinic derivatives reported to date are nonselective, the analogue of DG13, DG011, demonstrated a notable selectivity for ERAP2 (Temponeras et al., 2022). In MOLT-4 leukemia cell lines, DG011 caused a significant alteration in the immunopeptidome, leading to the detection of over 20% of peptides that were either new or significantly upregulated (Temponeras et al., 2022). Another ERAP2 selective inhibitor is Hydroxamic Acid Triazoles from KTGS, that in HEK cells, engages ERAP2 and inhibits SIINFEKL antigen presentation (Camberlein et al., 2022). The challenge of achieving selectivity among ERAP1, ERAP2, and other metalloproteases continues, particularly with allosteric inhibitors, that may provide a unique mode of action but require further investigation to understand their effects on immunopeptidome and T cell activation. Ongoing structural studies and diverse inhibitor development aim to improve specificity and potency while addressing the transport challenges of getting these compounds into cells.

Moreover, the inherent genetic variability of ERAP genes in humans necessitates careful selection of patient populations for clinical trials. The pharmacological profile of ERAP inhibitors has the potential for improved compliance and reduced side effects compared to biologics. In autoimmune diseases, these inhibitors may be used alone or in combination with existing therapies, potentially benefiting patients who have not responded to conventional treatments. In cancer immunotherapy, ERAP inhibitors could augment the effectiveness of existing therapies. Moreover, in the light of the multiple roles displayed by ERAPs in the maintenance of cellular homeostasis, it will be intriguing to determine whether ERAP1 and ERAP2 inhibitors also influence ER stress response, autophagy, cell migration and angiogenesis, potentially unveiling new therapeutic avenues for conditions characterized by dysregulated cellular stress responses and inflammation.

## 4 Conclusion

ERAP1 and ERAP2 emerge as versatile modulators in cellular systems, impacting a spectrum of processes that extend well beyond their traditional roles in antigen processing to influence cell migration, angiogenesis, UPR, and autophagy. ERAP1’s established links to endothelial migration and angiogenesis reveal its potential involvement in processes such as wound healing, vascular integrity, and tumor growth through interactions with VEGF signaling, cytoskeletal dynamics, and cell adhesion. By promoting or inhibiting these processes depending on the cellular context, ERAP1 could serve as a key regulator in the balance between normal tissue repair and pathological angiogenesis seen in conditions like cancer. ERAP2’s impact on UPR-driven autophagy further highlights a possible role in cellular resilience to internal stressors such as misfolded proteins and external challenges like oxidative stress, nutrient deprivation, and inflammatory cytokines. Such functions are particularly relevant in cancer progression, chronic inflammation, and autoimmune conditions, where cellular stress responses often become dysregulated.

Additionally, both ERAP1 and ERAP2 appear to participate in cytoskeletal organization and signaling pathways, suggesting they may act as integrative hubs that connect immune signaling with metabolic and stress-responsive networks. Polymorphisms and differential expression of ERAPs could further influence these varied cellular effects, modifying susceptibility to diseases like autoimmune disorders, cancer, and cardiovascular conditions. Together, these insights expand the paradigm of ERAP function and open possibilities for innovative therapies.
